# A novel trauma induced urethral stricture in rat model

**DOI:** 10.1038/s41598-024-55408-8

**Published:** 2024-03-15

**Authors:** Ziqiang Wu, Zhengyan Tang, Zhihuan Zheng, Shuo Tan

**Affiliations:** 1 Department of Urology, Xiangya Hospital of Central South University, Changsha, People’s Republic of China; 2 Department of Urology, Third Xiangya Hospital of Central South University, Changsha, People’s Republic of China; 3 Provincial Laboratory for Diagnosis and Treatment of Genitourinary System Disease, Changsha, People’s Republic of China

**Keywords:** Urethral stricture, Rat model, Cystostomy, Cystometry, Urethral fibrosis, Urology, Trauma, Urogenital diseases

## Abstract

Urethral stricture (US) is a longstanding disease, while there has not existed a suitable animal model to mimic the condition. We aimed to establish a trauma-induced US animal model to simulate this clinical scenario. A total of 30 rats were equally distributed into two groups, sham and US group. All rats were anesthetized with isoflurane and undergone cystostomy. In the US group, a 2 mm incision was made in the urethra and sutured to induce US. The sham group only make a skin incision on the ventral side of the anterior urethra. 4 weeks later, ultrasound and cystourethrography were performed to evaluate the degree of urethral stricture, pathological examinations were carried out to evaluate the degree of fibrosis. Urodynamic evaluation and mechanical tissue testing were performed to evaluate the bladder function and urethral tissue stiffness. The results showed that the urethral mucosa was disrupted and urethral lumen was stenosed in the US group. Additionally, the US group showed elevated bladder pressure, prolonged micturition intervals and increased tissue stiffness. In conclusion, the rat urethral stricture model induced by trauma provides a closer representation of the real clinical scenario. This model will significantly contribute to advancing research on the mechanisms underlying traumatic urethral stricture.

## Introduction

Urethral stricture (US) is a chronic condition characterized by fibrosis and narrowing of the urethral lumen, typically resulting from acute injury or iatrogenic interventions such as urethral instrumentation^1^. Although the use of grafts and flaps, such as penile skin, scrotal skin, buccal mucosa, lingual mucosa, and bladder mucosa, has significantly improved stricture-free rates after therapy^2–5^, surgical care for this condition remains challenging due to high recurrence rates and potential complications such as erectile dysfunction^6,7^. In order to improve the success rate of urethral reconstructive surgeries and find new ways to alleviate postoperative fibrosis and scar contraction, investigating the histological features and bladder pressure changes in animal models is crucial for US research.

However, as of yet, no standardized US animal model for research exists. Previous studies have utilized several methods to induce US^8,9^, including the electrocoagulation and drug induction methods in rat models^10,11^. Nonetheless, the previous studies regarding the evaluation of the mouse bladder and urethra lack systematicity, insufficiently replicate clinical damage, and fail to mimic clinical scenarios. In clinical settings, patients with urethral injuries typically undergo catheterization or urinary diversion promptly after trauma, whereas such simulations are absent in rat models.

Therefore, the purpose of this experimental study was twofold: first, to construct a simple and reproducible US rat model induced by trauma; second, to systematically investigate the imaging, histological and mechanical features and bladder pressure changes in trauma induced US rat model.

## Methods

### Experimental animals

A total of 30 male Sprague–Dawley rats (Slack Jingda Experimental Animal Corporation, Hunan, China), weighing approximately 300–350 g, were used in this study. The rats were housed in specific pathogen-free conditions in a 12-h cycle of alternating light and dark, with regular feeding and bedding replacement every 3–4 days. All experiments were conducted in accordance with animal care guidelines and approved by the Experimental Animal Welfare Center at Central South University, Hunan, China (202112630). Furthermore, all animal experiments were performed in accordance with relevant guidelines and regulations and reporting in the manuscript follows the recommendations in the ARRIVE guidelines 2.0.

### Study design and procedure

**Figure 1:**
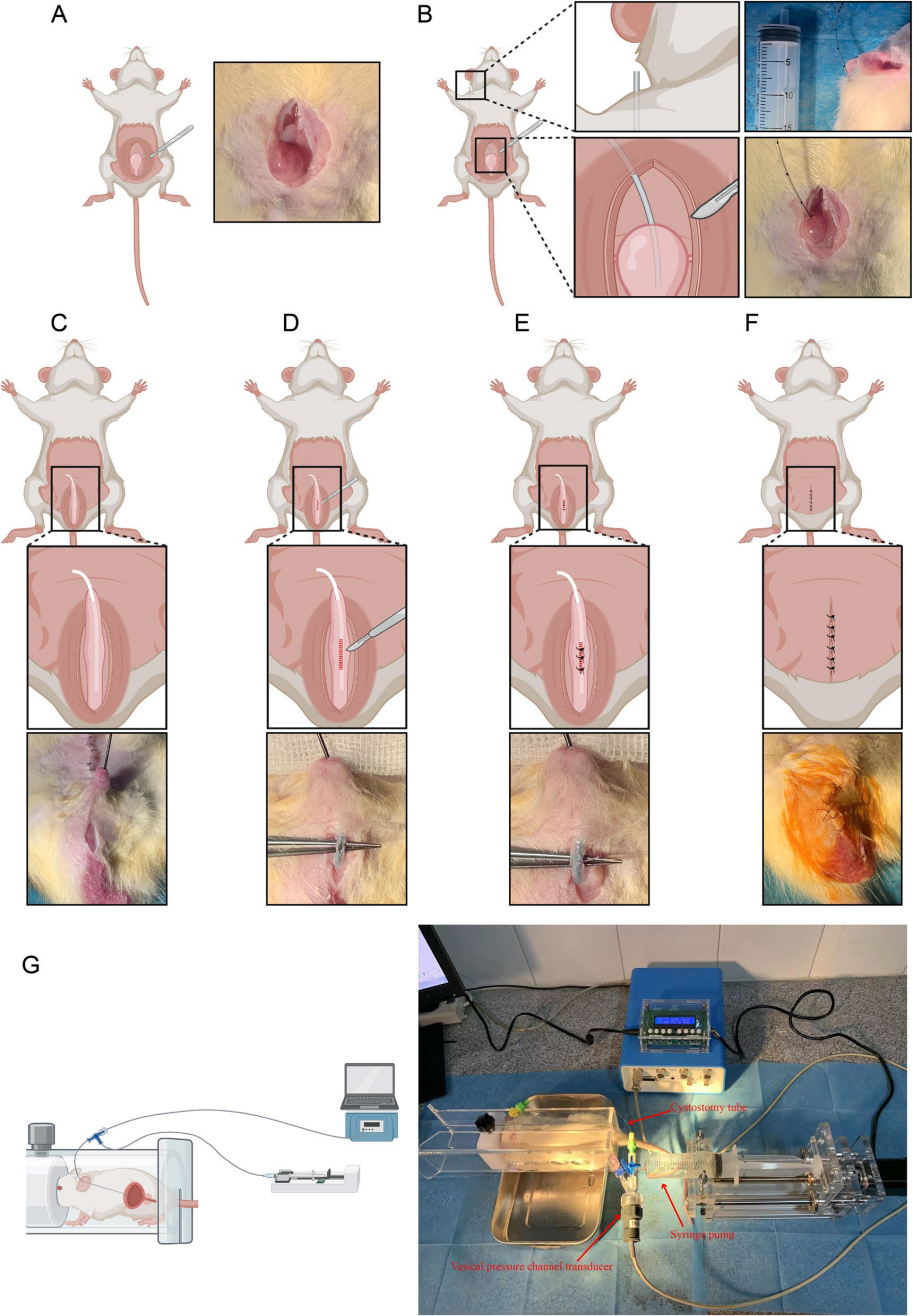
Surgical Procedures for Modeling. (**A**) Bladder Exposure. (**B**) Cystostomy for Rats. (**C**–**F**) Trauma-induced Urethral Stricture (US). (**G**) Cystometry for Rats under Consciousness.

30 SD male rats were randomly divided into two equal groups: (1) the sham group (cystostomy and sham surgery to the urethra, n = 15); (2) the experimental group (cystostomy and traumatic surgery to the urethra, n = 15). Meria et al.^12^ reported that incidence of urethral stricture after treatment was 50%. The sample size calculation for this study is based on relevant statistical formulas and the actual conditions of the experiment. Amoxicillin (50 mg/kg intraperitoneally) was administered 1 h before the surgical procedures to prevent infection. The rats were anesthetized with inhaled isoflurane and placed in the dorsal decubitus position. First, cystostomy was performed by making an incision in the abdomen to expose the bladder (Fig. 1A). A small incision was made in the bladder dome using a scalpel to insert an epidural catheter (1.0 mm diameter, Aoocn, Changyuan, China) into the bladder, and then the bladder incision was closed with 5-0 absorbable surgical suture (PGA suture) (Fig. 1B). The distal end of the epidural catheter protruded from the back of the rat's neck through a subcutaneous tunnel (Fig. 1B). The distal end of the epidural catheter can connect to a three-way stopcock to the vesical pressure channel transducer to perform cystometry. Next, to perform trauma-induced urethral stricture (US), the ventral penile skin was opened to expose the urethra, and a lubricated 0.03 inches polyurethane guidewire was inserted into the rat's urethra (Fig. 1C). A small incision (about 5 mm) was made through all layers of the urethral wall until the guidewire was visible (Fig. 1D). Finally, the urethra was misplaced sutured with 5-0 PGA suture, and the penile skin were sutured with 5-0 PGA suture (Fig. 1E,F). In the sham group, only an incision was made in the ventral penile skin, and no incision was made in the urethra.

### Urethral ultrasound and imaging assessment

The same sonographer at Feiyu Medical Imaging Center conducted urethral ultrasounds to assess the US model in vivo, while cystourethrography was also carried out at the same center. The bladder and proximal urethra of the US is visualized by injecting contrast agent through the cystostomy catheter, and the distal end is visualized by injecting contrast agent into urethral through an epidural catheter (1.0 mm diameter, Aoocn, Changyuan, China). The contrast agent was administered by the same experimenter, and the evaluations were conducted four weeks after modeling.

### Cystometric evaluation

Cystometry evaluation was conducted four weeks after surgery. To reduce the potential impact of anaesthetization on urodynamics, our cystometry measurements were conducted in the awake rats. During the cystometry process, the living rat is fixed in a rat holder, with a container underneath to catch the urine. We connected the cystostomy tube punctured from the back of the rat’s neck to a vesical pressure transducer (BL-420F, Taimeng Software Co., Ltd., Chengdu, China) and a syringe (Fig. 1G). Related study showed that the bladder capacity of a rat is about 1 ml^13^. It takes approximately 5 min for the bladder to reach full capacity and complete one micturition cycle, aligning with the urodynamic micturition cycle. In order to simulate the normal urination cycle of rats and adjust according to the actual situation of our experiment (rats will also produce urine during the experiment), we injected warm saline (37–38 °C) into the bladder at a rate of 8–10 ml/h through the syringe. The pressure transducer can record the pressure in the bladder and transmitted to the computer to form a real-time bladder pressure map. When the bladder is filled with urine and contracts, a pressure wave of bladder contraction can be recorded. Two to four voiding events were recorded for each rat in the sham and experimental groups to assess the voiding pressure of the bladder.

### Urethral tissue mechanical stiffness assessment

The rat urethral tissue was collected four weeks after surgery. The tissue was embedded in OCT and cut into slices of about 25 μm. Atomic force microscopy (AFM) was used to evaluate the urethral tissue mechanical stiffness. In touch mode, the force curve measurement was performed, and the Young's modulus of a 16 × 16 array of points was measured. Each sample was measured three times, and unqualified testing data were discarded. In the Asylum software, the Young’s modulus was automatically fitted to the needle entry curves of 256 force curves within the mapping range. The combined model is named Hertz model. The Gaussian distribution of stiffness (Young’s modulus) of the sample was obtained, and the average and standard deviation were taken for statistical analysis.

### Histology and immunofluorescence

Four weeks after surgery, the urethral tissue was excised and fixed in 4% paraformaldehyde overnight, followed by routine histological procedures. The sections (4 μm) were stained with hematoxylin and eosin (H&E), Masson's trichrome (MTS), and immunofluorescence (IF) to evaluate the morphology and fibrosis of the urethral tissue. IF was performed using anti-α-SMA antibody (1:200, ab7817, Abcam), anti-vimentin antibody (1:200, ab8069, Abcam), anti-COL1 antibody (1:200, ab254113, Abcam), and anti-CD31 antibody (1:200, 3528, CST) according to the instructions.

### Western blot

Four weeks after surgery, urethral tissues were harvested and used for extracting total protein. The protein concentration was determined using the BCA kit (NCM Biotech, Suzhou, China). The protein was separated and transferred via cataphoresis using 10% SDS-PAGE gels electrophoresis and PVDF membranes. The PVDF membranes were then incubated overnight at 4 °C with anti-α-SMA antibody (1:2000, ab7817, Abcam), anti-vimentin antibody (1:2000, ab8069, Abcam), anti-COL1 antibody (1:2000, ab260043, Abcam), anti-fibronectin (1:2000, ab2413, Abcam), anti-GAPDH antibody (1:3000, ab8245, Abcam), and anti-Tubulin (1:2000, 5335, CST). After washing with PBST, blots were detected by secondary antibody and visualized by the ECL assay.

### Gene expression evaluation by reverse transcription-quantitative PCR (RT-qPCR)

Total RNA was extracted from urethral tissue four weeks after surgery using RNAiso Plus reagent (Takara Bio Inc., Otsu, Shiga, Japan), and cDNA was synthesized using the PrimeScript RT reagent kit (Takara Bio Inc. Otsu, Shiga, Japan). The primers used in this study are shown in Supplementary S1. The gene expression in urethral tissue was then verified by RT-qPCR methods. The data is expressed as a ratio of the sham gene *Gapdh*.

### Statistical analysis

All data were expressed as mean ± standard deviation based on the results of three independent repeated experiments. Student's t-test was used to analyze the variance between two groups, and the significance level was set at *p* < 0.05. Prism 8.0 (GraphPad Software, San Diego, CA, USA) was used to perform the statistical analyses.

## Results

### Animals

After modeling, 2 out of 15 (13.3%) rats in the US group died due to incision infection and intra-abdominal infections, while 1 out of 15 (6.7%) rats from the same group developed a urinary fistula. No rats in the sham group experienced these complications. The incidence of fistula tube displacement was 20.0% (3/15) in the sham group and 13.3% (2/15) in the US group (Table 1).

**Table 1:** Complications after surgery.

	Mortality rate (%)	Incision infection rate (%)	Fistula tube displacement rate (%)	Urinary fistula rate (%)	Intra-abdominal infection rate (%)
Control	0	0	20	0	0
US	13.3	13.3	13.3	6.7	13.3

*US* urethral stricture.

### Urethral ultrasound and imaging assessment

**Figure 2:**
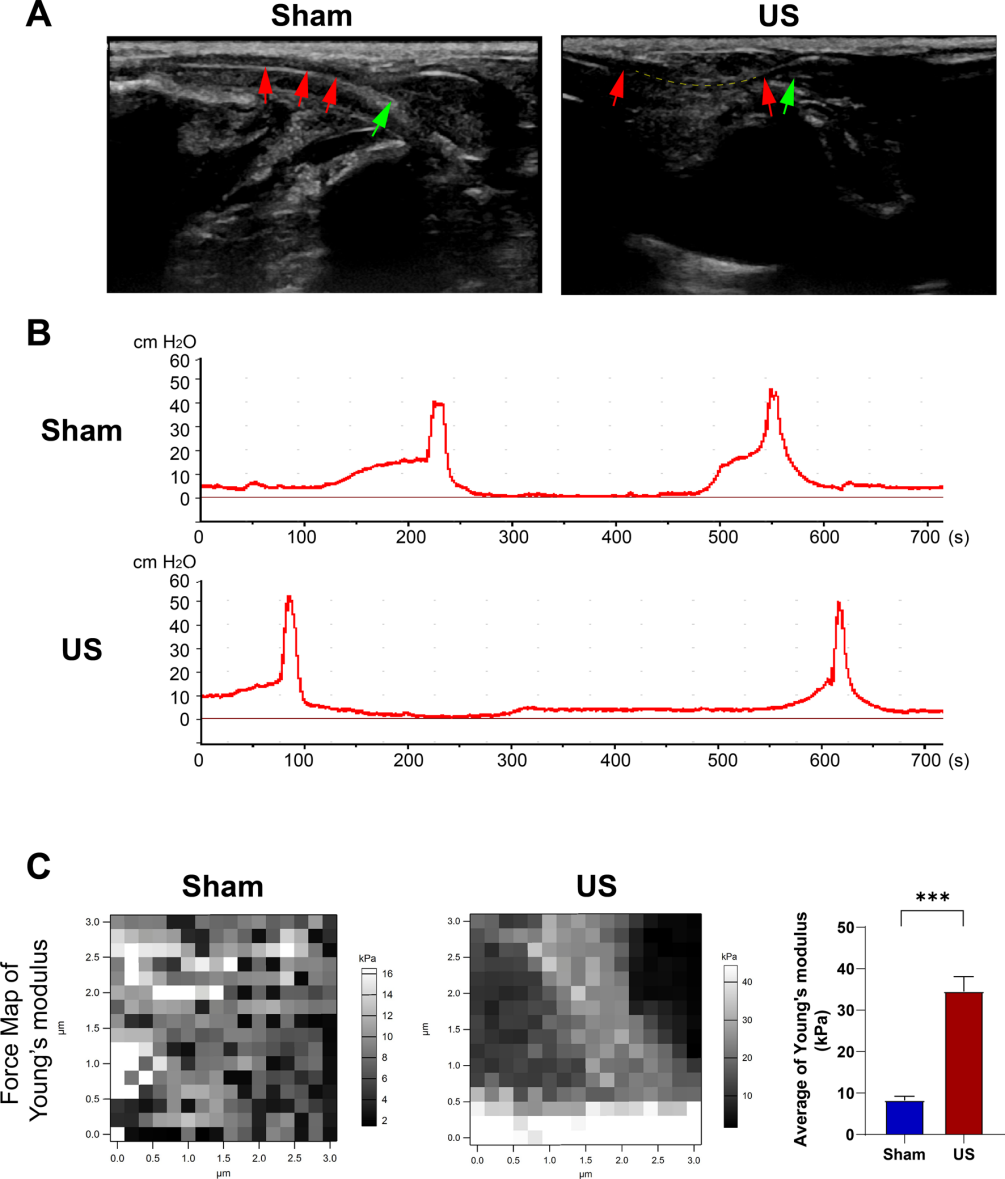
(**A**) Representative ultrasound Images of urethras in a control rat and a rat with urethral stricture (US). Red arrows indicate urethral lumen, green arrow represents bulbar urethra, and yellow dotted line depicts the position of US. (**B**) Representative urodynamic tracing from rats with bladder catheter. BP: Baseline Pressure (the lowest pressure between two voids); TP: Threshold Pressure (pressure shortly before void initiation); MP: Maximum Pressure (highest pressure during void). (**C**) Representative Young's Modulus Plot for assessing tissue matrix stiffness.

**Figure 3:**
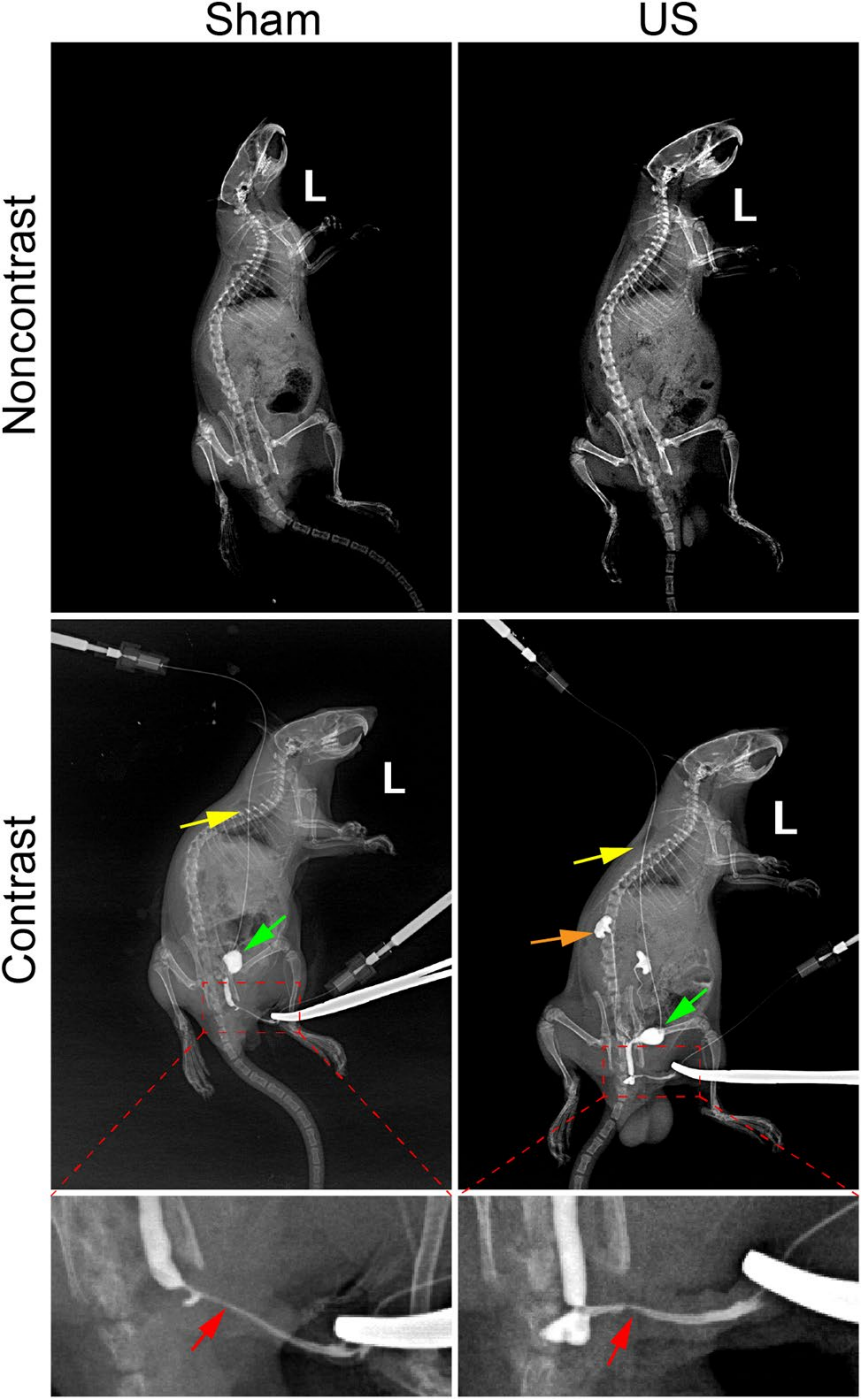
Representative cystourethrography images of a control rat and a rat with urethral stricture (US). Red arrows indicate urethral lumen (the US rat exhibited defects in contrast filling), green arrows represent bladder, orange arrow represents kidney and yellow arrows represents the cystostomy tube.

The B-ultrasound of the urethra showed that the anterior urethral lumen in the sham group was complete and unobstructed, while the anterior urethral lumen was visibly disrupted in the US group (Fig. 2A). The urethra (red arrow) and bladder (green arrow) can be fully visualized after injection of contrast agent (Fig. 3). In the US group, the rat exhibited urethral filling defects (red arrow) and bilateral vesicoureteral reflux (orang arrow), whereas the rat in the sham group had an unobstructed urinary tract and a fully developed urethral lumen (red arrow) (Fig. 3).

### Cystometry

Compared to the sham group, the cystometry results showed a 25.1% increase in maximum pressure (MP) (42.4 ± 2.53 vs. 53.05 ± 2.94,* p* < 0.01) and a 49.2% extension in micturition interval (MI) (347.04 ± 23.28 vs. 517.65 ± 17.92, *p* < 0.001) in the US group. The US group exhibited a faster peak in bladder pressure curve and threshold pressure (TP) during urination. However, there were no significant differences in basal pressure (BP) (6.4 ± 0.90 vs. 7.46 ± 1.08) and TP (14.8 ± 1.56 vs. 16.01 ± 1.34) between the two groups (Table 2) (Fig. 2B).

**Table 2:** Urodynamic parameters of the bladder.

	MP (H_2_0 cm)	BP (H_2_0 cm)	TP (H_2_0 cm)	MI (s)
Control	42.4 ± 2.53	6.4 ± 0.90	14.8 ± 1.56	347.04 ± 23.28
US	53.05 ± 2.94**	7.46 ± 1.08	16.01 ± 1.34	517.65 ± 17.92***

*MP* maximum pressure, *BP* basal pressure, *TP* threshold pressure, *MI* micturition interval, *US* urethral stricture (**p < 0.01, ***p < 0.001 versus control group).

### Matrix stiffness assessment

The white high-stiffness regions in the US group were concentrated in sheets, and the stiffness of the urethral tissue increased 316.9% (8.30 ± 0.91 vs. 34.6 ± 3.50, *p* < 0.001) when compared to the sham group, four weeks after surgery (Fig. 2C).

### Histology and immunofluorescence

**Figure 4:**
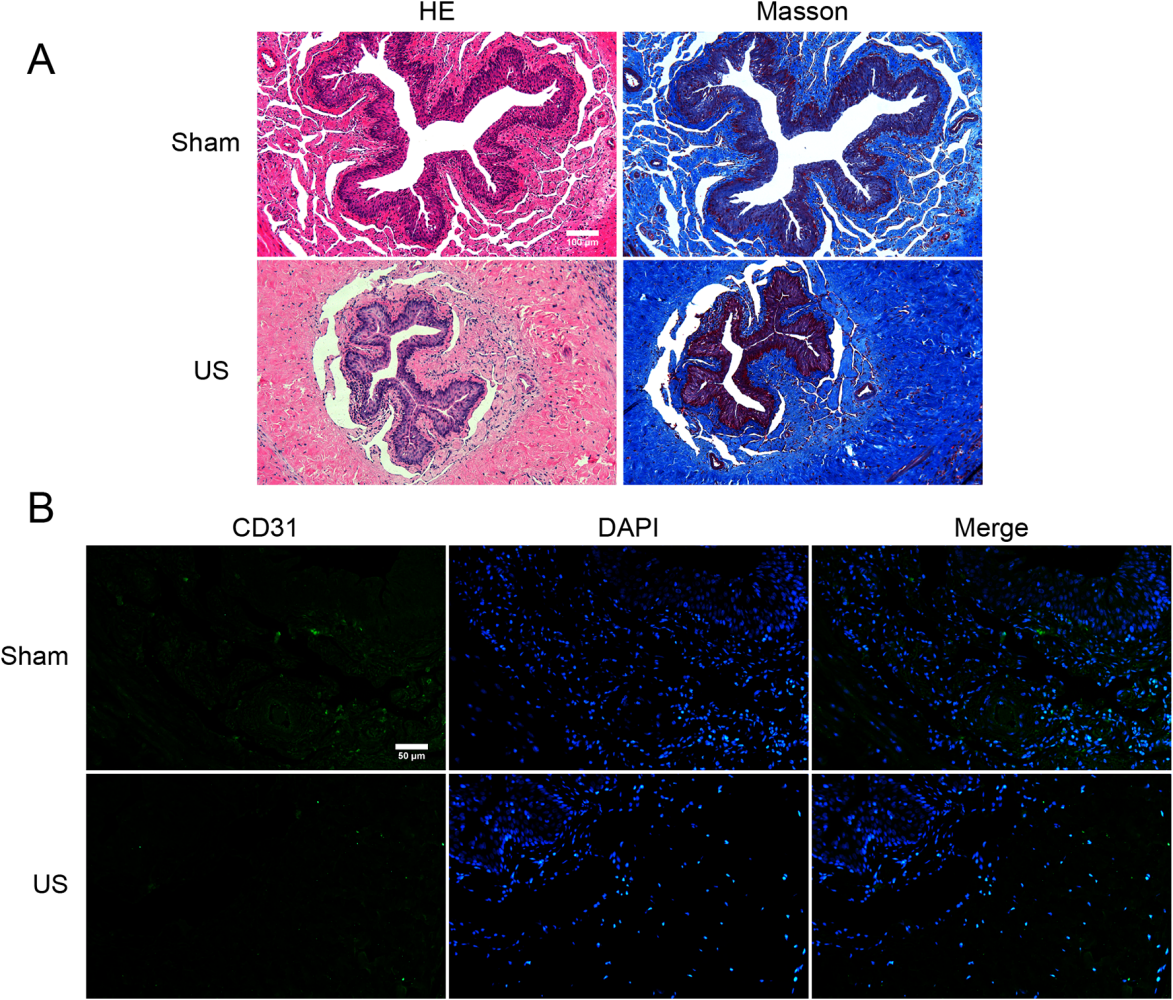
Histology: (**A**) Representative HE and Masson’s trichrome staining of urethra. The US rat showed narrowed urethral lumen and deposited collagen. (**B**) Representative Immunofluorescence of CD31 staining showed reduced blood vessels around the urethra in US Rat.

**Figure 5:**
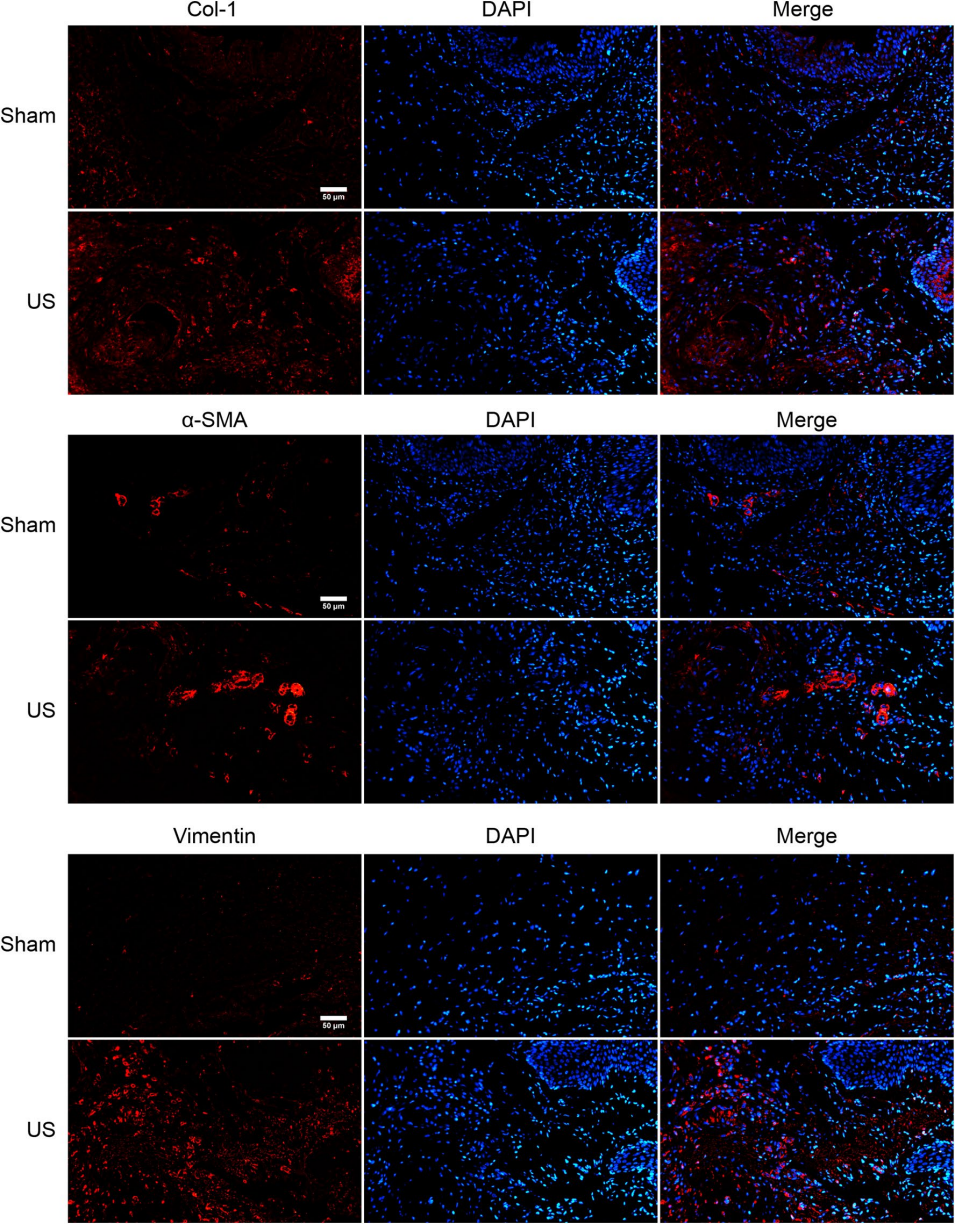
Representative immunofluorescence. The fibrosis-related markers of Col-1, α-SMA, and Vimentin increased in US Rats.

In the US rat urethral specimens, the HE and Masson urethral sections showed a narrowed urethral lumen and damaged urothelial cells. Meanwhile, the muscular layer showed that collagen bundles invade and envelops muscle fibers (Fig. 4A). The urethral lumen in the sham group was normal, and the collagen fibers were arranged neatly (Fig. 4A). The immunofluorescence analysis of CD31 staining showed that blood vessels around the urethra reduced significantly in the US group (Fig. 4B). Additionally, the immunofluorescence results indicated increased expression of fibrosis-related markers such as collagen-1, vimentin, and α-SMA in US rats (Fig. 5).

### Western blot and gene expression

**Figure 6:**
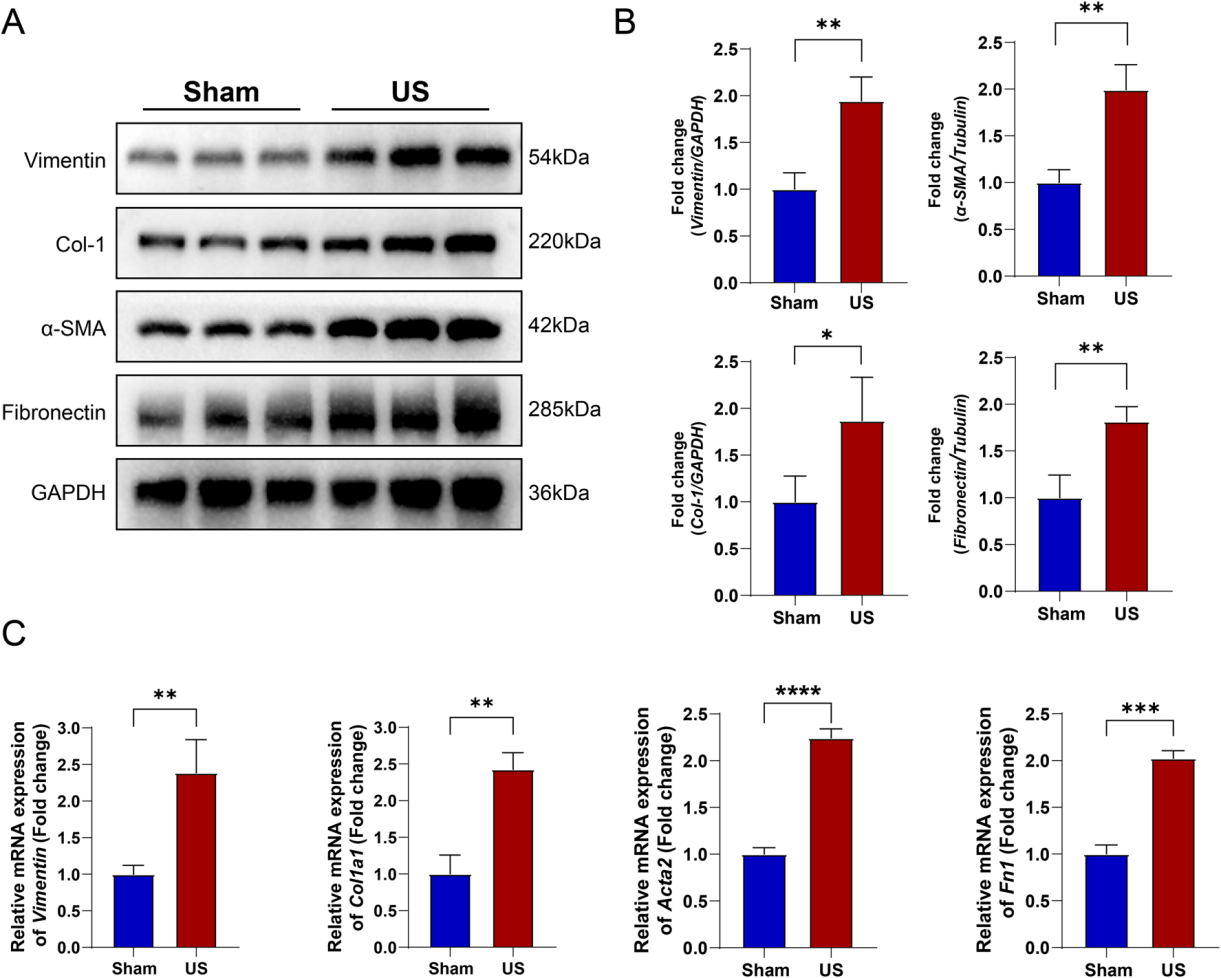
Expression of fibrosis markers in control rats and US Rats: A-B) Western blot analysis demonstrating up-regulation of Vimentin, Col-1, α-SMA, and Fibronection in US rats (*p < 0.05; **p < 0.01). C) qPCR analysis demonstrating relative mRNA expression of *Col1a1*, *Acta2*, *Vimentin*, and *Fn1* compared to GAPDH (*p < 0.05; **p < 0.01, ***p < 0.001, ****p < 0.0001).

To confirm urethral fibrosis, we evaluated the expression of fibronectin, collagen-1, vimentin, and α-SMA by Western blot and RT-qPCR analysis. Western blot analysis revealed that the protein contents of fibronectin, collagen-1, vimentin, and α-SMA were significantly higher in the urethral tissues of the US group (*p* < 0.05; Fig. 6A,B). In addition, the RT-qPCR analysis confirmed that the genes of *Col1a1*, *Acta2*, *Vimentin*, and *Fn1* were highly expressed in the US group compared with the sham group (Fig. 6C).

## Discussion

This study reports a novel US rat model with cystostomy. This method is suitable for rats to inducing US due to its high success rate and low postoperative complication rate. Previous studies have reported similar methods in other animal models, such as a US rabbit model created by excising 0.8 × 2 cm of the ventral urethra and then suturing the defect by primary repair^14^, and the induction of US in a porcine model by ligation of the urethral^15^. However, few researchers have used this method to create a US model in rats, and a standardized rat US model induced by trauma similar to clinical trauma patients is not available.

Several methods have been attempted in the past to induce US in rats, including using TGF-β1 to induce US in a rat model^16,17^, but this method differs from trauma-induced US in clinical practice. Additionally, it is difficult to control the dose of TGF-β1 injected into the urethra. Sangkum et al.^16^ reported using a dose of 5–25 μg to induce urethral stricture, while Castiglione et al.^17^ used a dose of 1 μg. Krane et al.^10^ reported the induction of US in rats using electrocautery, but they did not perform cystostomy for the rats, which can cause bladder and upper urinary tract dysfunction if obstruction is too long or retention occurs. In our modeling method, a secondary surgery to perform cystometry is not necessary, which can reduce secondary trauma and complications to the rats.

The ultrasound analysis showed that the urethra lumen of the US group was incomplete and interrupted, which is similar to previous studies^16–18^. Furthermore, the imaging assessment uncovered that the rat in the US group displayed urethral filling defects and ureteral reflux, which is in accordance with clinical practice. It is commonly known that retrograde urethrography (RUG) with or without voiding cystourethrography (VCUG) can be utilized in clinical practice to identify the location of a stricture in the urethra, the length of the stricture, and the extent of lumen narrowing^1^. Although cystourethrography has been employed to evaluate the US in animal models like rabbits and porcine^8,15^, there are limited studies in the current literature regarding the use of cystourethrography in rat models. We developed this approach in rats and demonstrated the formation of US in a rat model via cystourethrography.

Cystometry analysis revealed a significant increase in MP and MI in US rats compared to the sham group, but no differences in BP and TP were detected between the two groups. These results were different from other studies, where US rats displayed no differences in MP, but increased BP, TP, and reduced MI compared to the sham group^17^. We speculate that the observed differences in MI and MP results may be due to the severity of our urethral injury and the degree of obstruction, which may have been greater than that induced by TGF-β1. As a result, the US group required higher pressure and greater capacity for urination, resulting in longer MI and higher MP values. Additionally, we performed cystostomy when rat modeling, which helped preserve bladder function and prevent bladder decompensation. This likely contributed to the lack of significant differences in BP and TP between the sham and US groups. Our results were consistent with other studies of lower urinary tract obstruction^19,20^. Seki et al.^19^ demonstrated that max detrusor pressure was decreased after relieving the lower urinary tract obstruction in humans, and Chen et al.^20^ showed that MP was significantly increased after obstructing the bladder outlet for two weeks in mice. We suppose that short-term partial bladder outlet obstruction can be compensated by the contraction function of the bladder.

Previous studies have demonstrated that tissue stiffness-based sonoelastography are safe and potentially effective methods for evaluating bulbar urethral strictures and spongiform fibrosis in humans^21,22^. Now, we were able to precisely measure the stiffness of rat urethral tissue using AFM after obtaining the urethral tissue. We found that the matrix stiffness of urethral tissue was greater in the US group than the sham group. This is consistent with our experience in clinical practice, where the urethral scar is hard to cut during urethral reconstructive surgery due to dense scar tissue. Besides, some basic research about matrix stiffness and fibrosis suggests that increased ECM substrate stiffness can interact with fibroblasts and cause a profibrotic vicious cycle^23,24^. More and more literature reports that increased matrix stiffness is strongly associated with progression of fibrotic disease, such as skin fibrosis^25^, liver fibrosis^26^, pulmonary fibrosis^27^ and renal fibrosis^28^. Our current study confirms this point of view in urethral fibrosis, but the regulation mechanism needs further study.

In the course of our research, we found that local histopathological changes in rat urethra following urethral injury had stabilized by 4 weeks, in comparison to the observations at 2 and 8 weeks. Consequently, our evaluation focused on urethral tissue in rats 4 weeks post-surgery. The histology assessment revealed that the urethral lumen in the US group rats was damaged, and extensive collagen was deposited in the submucosal connective tissue. Fibrosis-related markers (fibronectin, collagen-1, vimentin, and α-SMA) were increased in US rats in WB experiments. Additionally, the expression of fibrosis-related mRNA (*Col1a1, Acta2, Vimentin, Fn1*) was upregulated in the US group, consistent with previous studies^11,29^. The results validate the pathological process of trauma-induced urethral fibrosis^1^. The IF analysis of CD31 showed that blood vessel density around the urethra was reduced in US rats compared to the sham group. Ischemia is known to contribute to the progression of bladder fibrosis^30^, and the urethra is more sensitive to ischemia than the bladder^31^.

This study has some potential limitations. Firstly, we know that urodynamics and bladder function are primarily influenced by the human bladder’s mucosa layer (providing a barrier and protection) and detrusor muscle layer (providing micturition, detrusor pressure, and compliance). Both them are critical tissue structures. Meanwhile, in rodents, such as rats, the bladder only comprises mucosal layer, lamina propria, detrusor layer, and serosal layer. It's worth noting that the absence of muscularis mucosae in rat results in a lack of high similarities in anatomy and tissue structure when compared to humans. This is an aspect that requires further consideration. Secondly, the urethral was not assessed by urethrocystography due to its small lumen and the stricture length was not accurately measured. Thirdly, we only detected the matrix stiffness of the urethra, while the regulatory mechanism of stiffness affecting urethral fibrosis was not studied. Further basic research is required to confirm our findings and reveal the regulatory mechanism. However, despite these limitations, our study provides a possible animal US model that is similar to trauma-induced US patients.

## Conclusion

In summary, trauma can effectively induce a rat model of urethral stricture and this method can accurately simulates the disease progression in clinical settings. This new model demonstrated the feasibility of urethral diversion in the acute phase after lower urinary tract obstruction rats and it can be used to do urodynamic and imaging evaluation in live rats.

### Supplementary Information

Supplementary Information 1.

Supplementary Information 2.

## Data Availability

All relevant data are included in the manuscript. Materials, data, and protocols described within the paper are available upon reasonable request to the corresponding authors.
