# Supplementary material for: A novel trauma induced urethral stricture in rat model
Source: Sci Rep. 2024 Mar 15;14:6325. doi: 10.1038/s41598-024-55408-8 (PMC10943079; doi:10.1038/s41598-024-55408-8)
Supplement: Supplementary file 2 — Supplementary Information 2. [file 41598_2024_55408_MOESM2_ESM.docx]

**Supplementary S1**

*Col1a1*

Forward 5'-TGTTGGTCCTGCTGGCAAGAATG-3'

Reverse 5'- GTCACCTTGTTCGCCTGTCTCAC-3'

*Acta2*

Forward 5'- AGACCCTCTTCCAGCCATCT-3'

Reverse 5'- CCCCGAGAGGACGTTGTTAG-3'

*Vimentin*

Forward 5'- TGAATGACCGCTTCGCCAACTAC-3'

Reverse 5'- CAACTCCCTCATCTCCTCCTCGTAG-3'

*Fn1*

Forward 5'- AGGCACAAGGTCCGAGAAGAGG-3'

Reverse 5'- GGTCAAAGCATGAGTCATCCGTAGG-3'

*Gapdh*

Forward 5'- ACGGCAAGTTCAACGGCACAG-3'

Reverse 5'- CGACATACTCAGCACCAGCATCAC-3'

**Supplementary S2**

| **Young's modulus (kPa) used for analysis** | | |
| --- | --- | --- |
|  | Sham | US |
|  | 7.30 | 35.30 |
|  | 8.50 | 30.80 |
|  | 9.10 | 37.70 |
| **Mean** | 8.30 | 34.60 |
| **Standard deviation** | 0.92 | 3.50 |

| **Raw data for analysis of western blot** | | | | | | |
| --- | --- | --- | --- | --- | --- | --- |
|  | **Sham** | | | **US** | | |
| **Vimentin** | 45729.40 | 41539.14 | 37075.84 | 60831.14 | 89742.67 | 109464.23 |
| **GAPDH** | 81752.13 | 104579.38 | 82633.67 | 76994.60 | 98435.43 | 106245.16 |
| **Vimentin/GAPDH** | 0.56 | 0.40 | 0.45 | 0.79 | 0.91 | 1.03 |
| **Homogenization** | 1.19 | 0.85 | 0.96 | 1.69 | 1.95 | 2.20 |
|  |  |  |  |  |  |  |
| **COL-1** | 48710.91 | 44901.51 | 55520.27 | 83885.40 | 97672.06 | 104912.55 |
| **GAPDH** | 66773.92 | 107055.55 | 102378.14 | 101317.24 | 98569.89 | 78254.87 |
| **COL-1/GAPDH** | 0.73 | 0.42 | 0.54 | 0.83 | 0.99 | 1.34 |
| **Homogenization** | 1.29 | 0.74 | 0.96 | 1.47 | 1.76 | 2.38 |
|  |  |  |  |  |  |  |
| **SMA** | 43644.81 | 57820.05 | 48851.02 | 83839.06 | 108335.79 | 98759.01 |
| **Tubulin** | 101181.49 | 104149.28 | 108360.62 | 103753.49 | 107508.02 | 94234.62 |
| **SMA/Tubulin** | 0.43 | 0.56 | 0.45 | 0.81 | 1.01 | 1.05 |
| **Homogenization** | 0.90 | 1.16 | 0.94 | 1.69 | 2.10 | 2.19 |
|  |  |  |  |  |  |  |
| **Fibronectin** | 44726.53 | 61369.79 | 70812.74 | 89593.43 | 85462.60 | 95815.09 |
| **Tubulin** | 102579.89 | 102838.23 | 98684.87 | 94052.42 | 76246.52 | 86855.25 |
| **Fibronectin/Tubulin** | 0.44 | 0.60 | 0.72 | 0.95 | 1.12 | 1.10 |
| **Homogenization** | 0.75 | 1.02 | 1.23 | 1.63 | 1.92 | 1.89 |

| **Raw data for analysis of RT-qPCR** | | | |
| --- | --- | --- | --- |
| **Sample Name** | **Target Name** | **CT value** | **Gapdh mean CT value** |
| **Sham** | col1 | 20.48 | 18.65 |
| **Sham** | col1 | 20.96 | 18.65 |
| **Sham** | col1 | 21.18 | 18.65 |
| **US** | col1 | 19.57 | 18.77 |
| **US** | col1 | 19.71 | 18.77 |
| **US** | col1 | 19.84 | 18.77 |
| **Sham** | fn | 19.32 | 18.65 |
| **Sham** | fn | 19.46 | 18.65 |
| **Sham** | fn | 19.60 | 18.65 |
| **US** | fn | 18.63 | 18.77 |
| **US** | fn | 18.55 | 18.77 |
| **US** | fn | 18.52 | 18.77 |
| **Sham** | sma | 20.99 | 18.65 |
| **Sham** | sma | 21.17 | 18.65 |
| **Sham** | sma | 21.01 | 18.65 |
| **US** | sma | 20.03 | 18.77 |
| **US** | sma | 19.95 | 18.77 |
| **US** | sma | 20.07 | 18.77 |
| **Sham** | vim | 20.65 | 18.65 |
| **Sham** | vim | 20.98 | 18.65 |
| **Sham** | vim | 20.68 | 18.65 |
| **US** | vim | 19.89 | 18.77 |
| **US** | vim | 19.36 | 18.77 |
| **US** | vim | 19.71 | 18.77 |
