# Supplementary material for: A novel trauma induced urethral stricture in rat model
Source: Sci Rep. 2024 Mar 15;14:6325. doi: 10.1038/s41598-024-55408-8 (PMC10943079; doi:10.1038/s41598-024-55408-8)

Dear Editor,

Thank you for providing valuable suggestions on our manuscript. We have incorporated your feedback and submitted the original figures of Western Blot (WB) along with the results from three repeated experiments.

In the WB experiments, our approach involves cutting the membrane. This is aimed to improve the efficiency of the experiments.

We appreciate your continued guidance and look forward to any further recommendations you may have.

Sincerely,
Ziqiang Wu
Xiangya hospital, Central South University
xy-ziqiang@csu.edu.cn.

Collagen 1

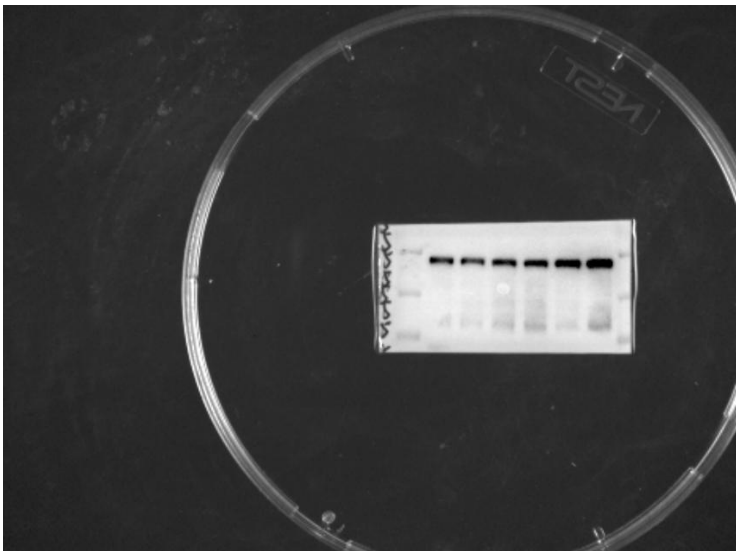

GAPDH

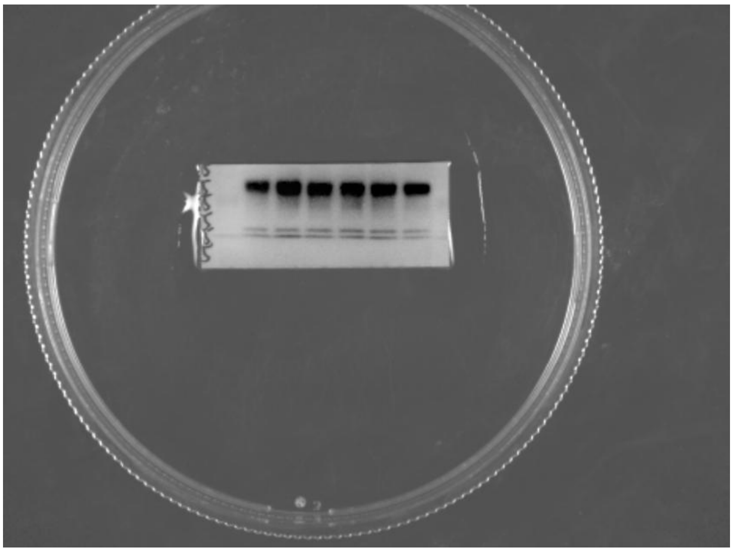

Fibronectin

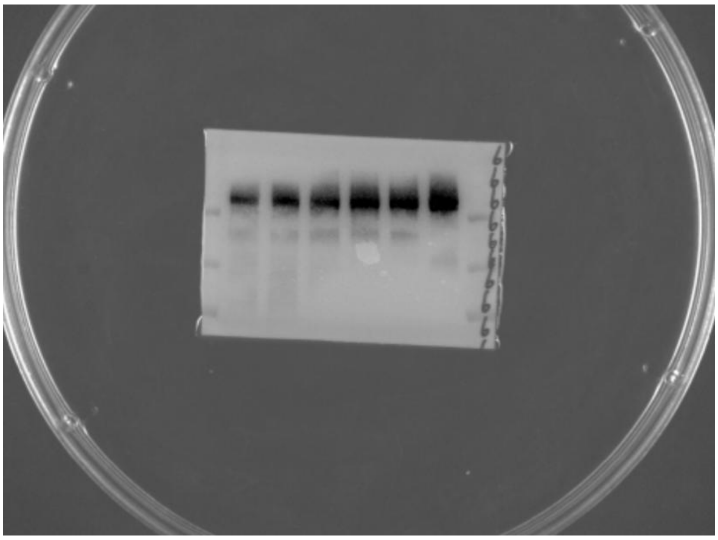

Tubulin

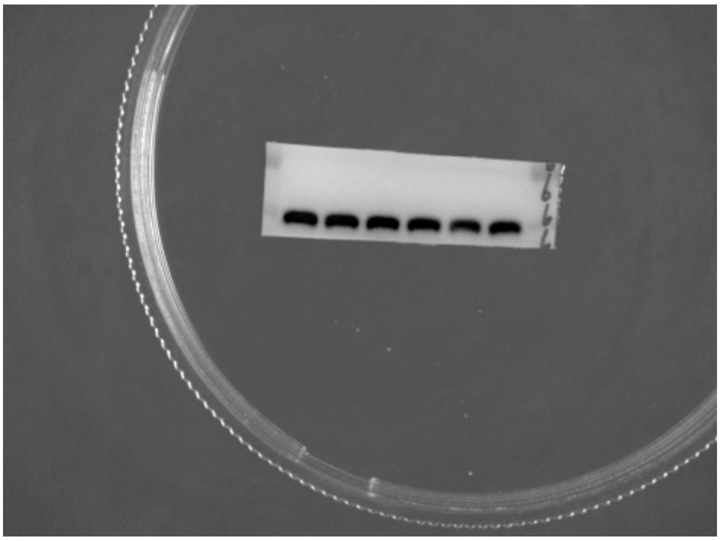

α-SMA

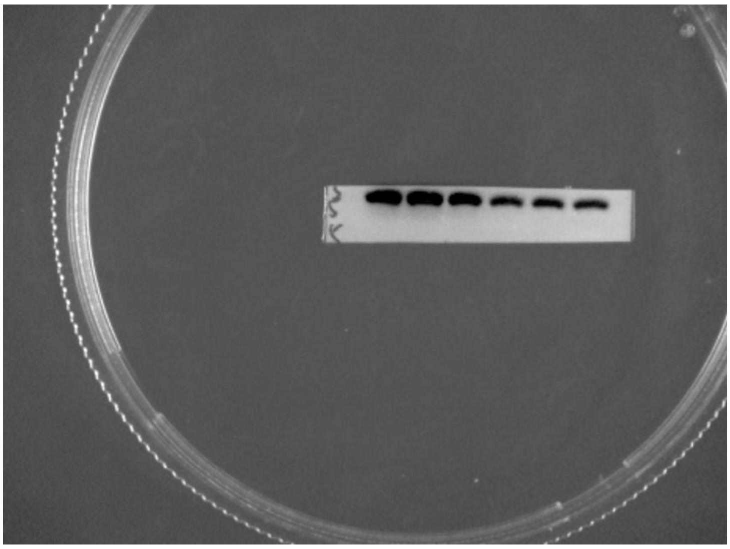

Tubulin

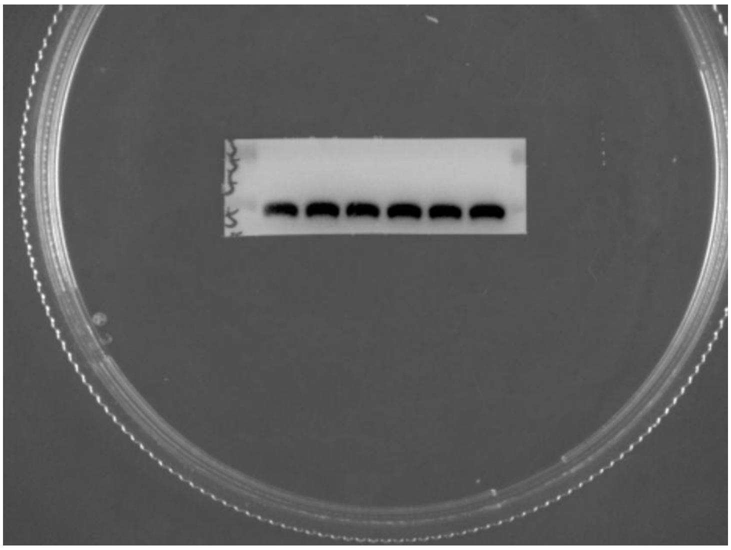

Vimentin

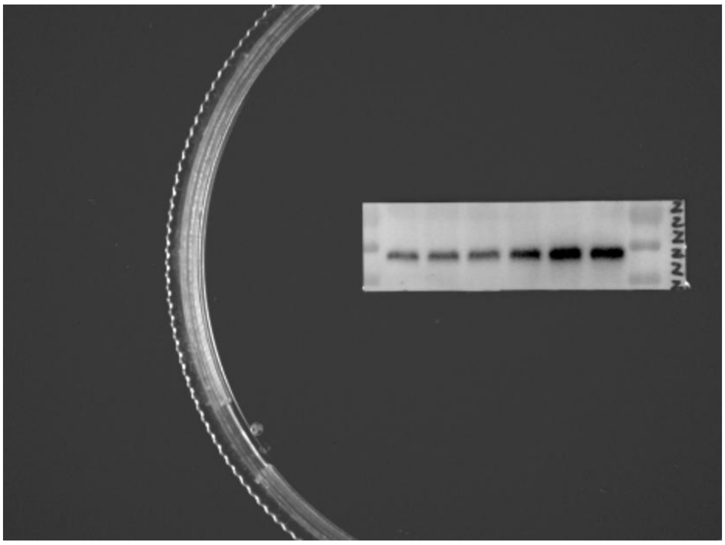

GAPDH

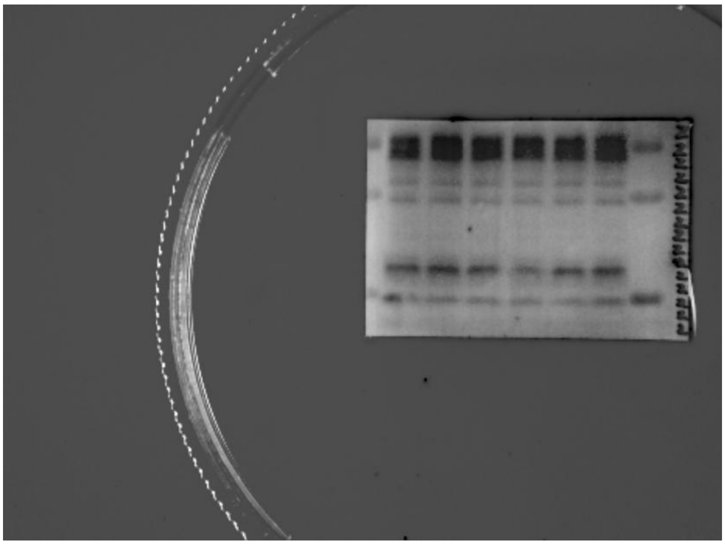

Replicate 1

Collagen 1

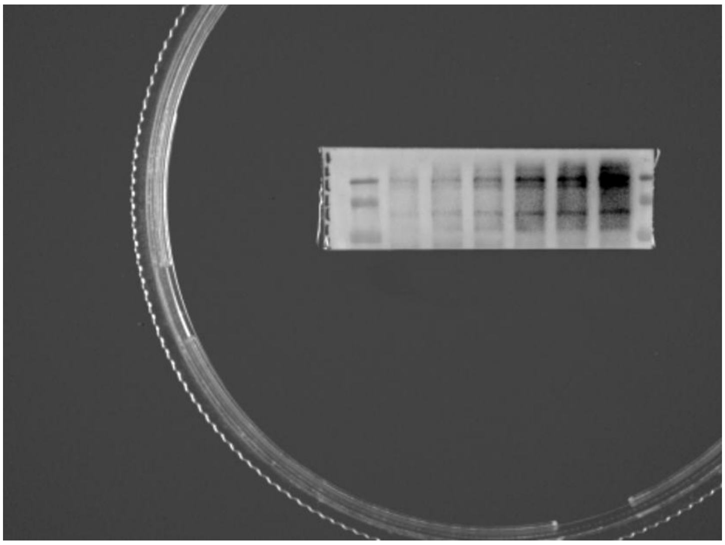

GAPDH

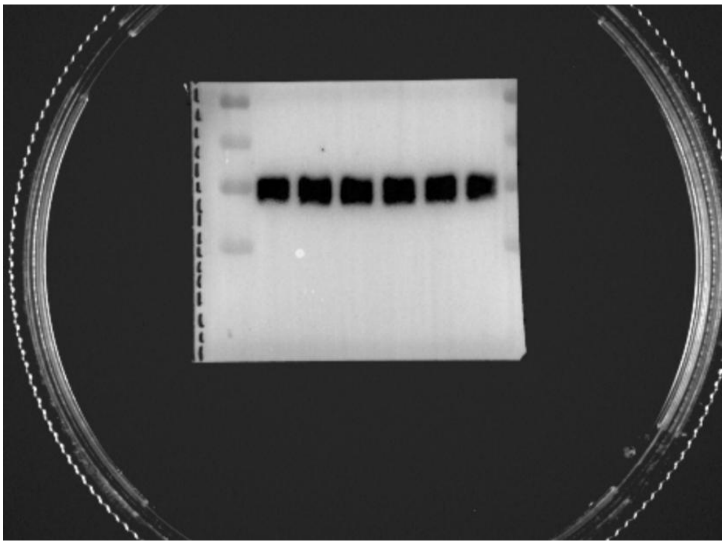

Fibronectin

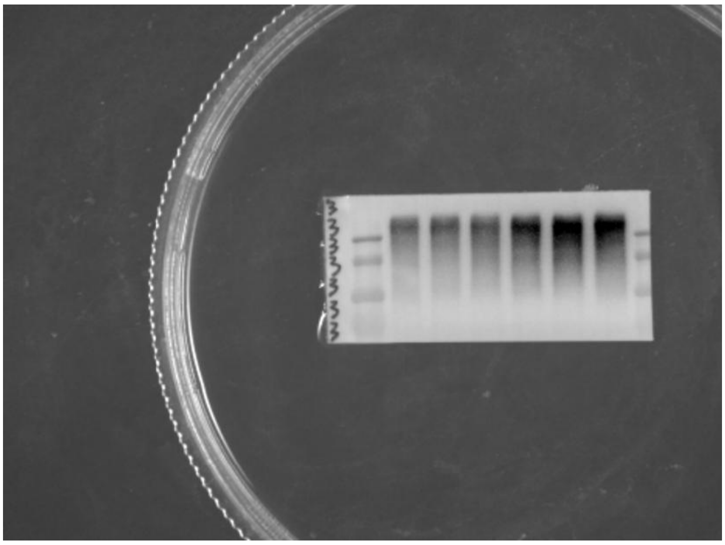

GAPDH

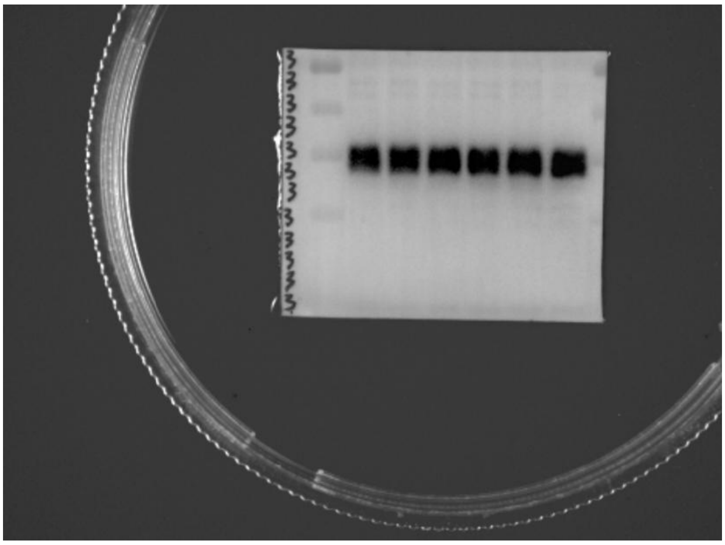

α-SMA

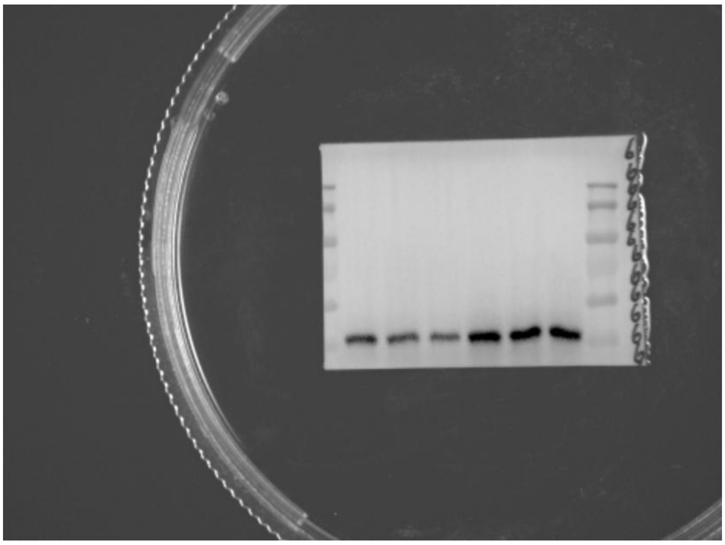

GAPDH

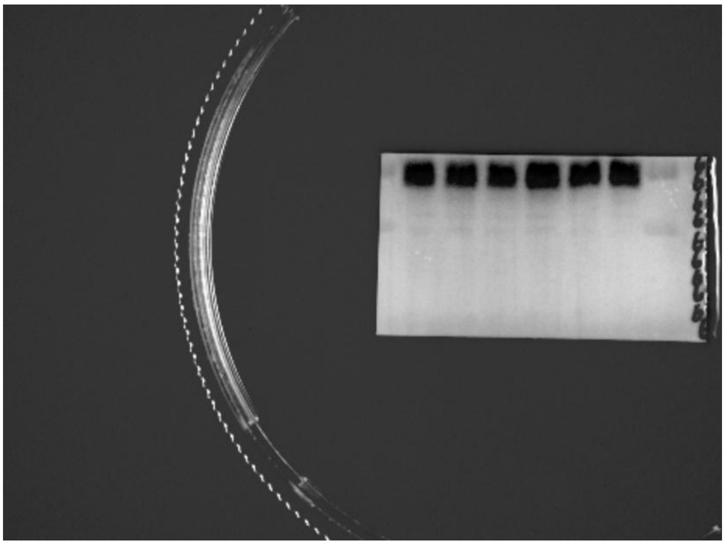

Vimentin

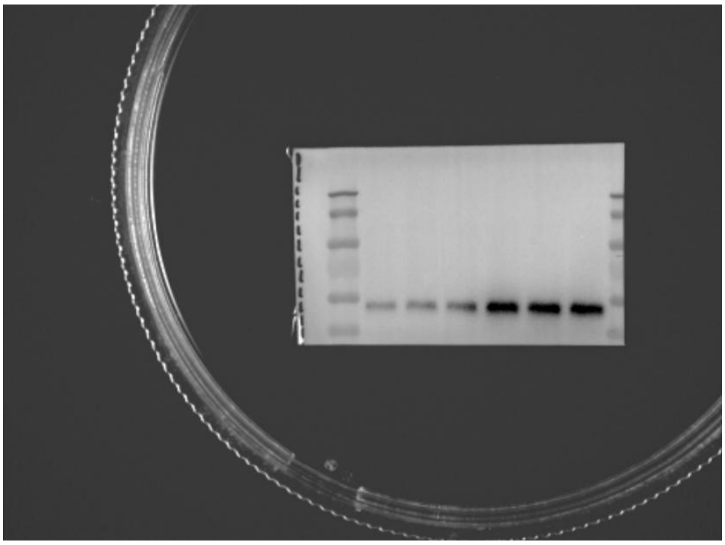

GAPDH

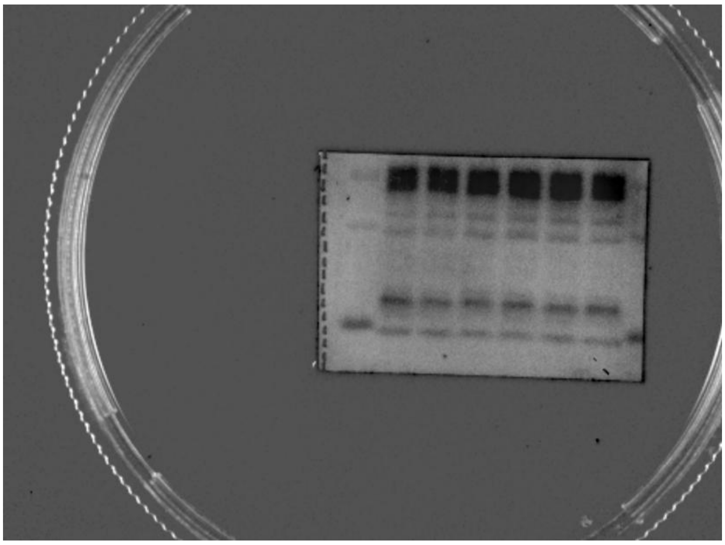

Replicate 2

Collagen 1

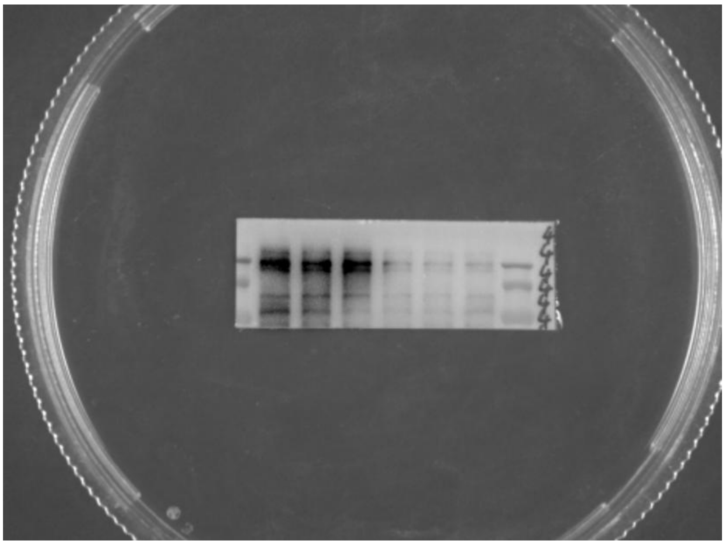

GAPDH

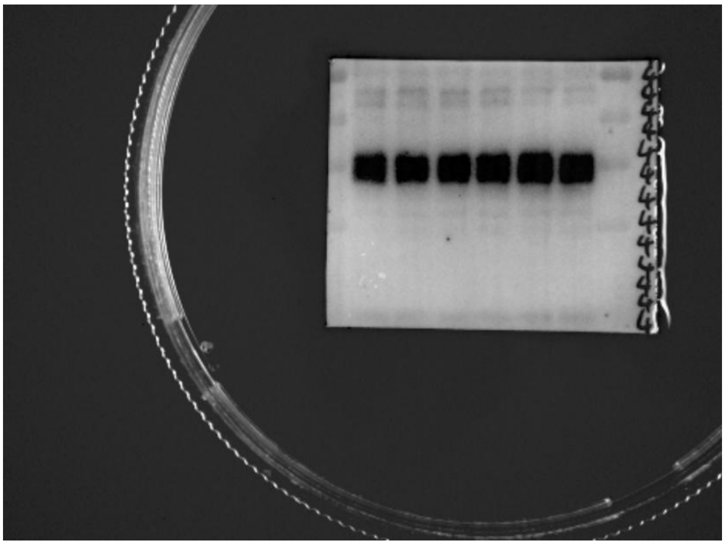

Fibronectin

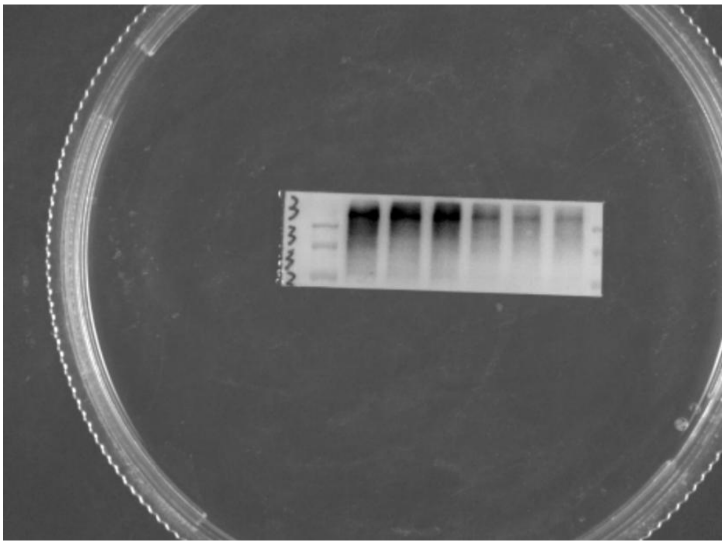

GAPDH

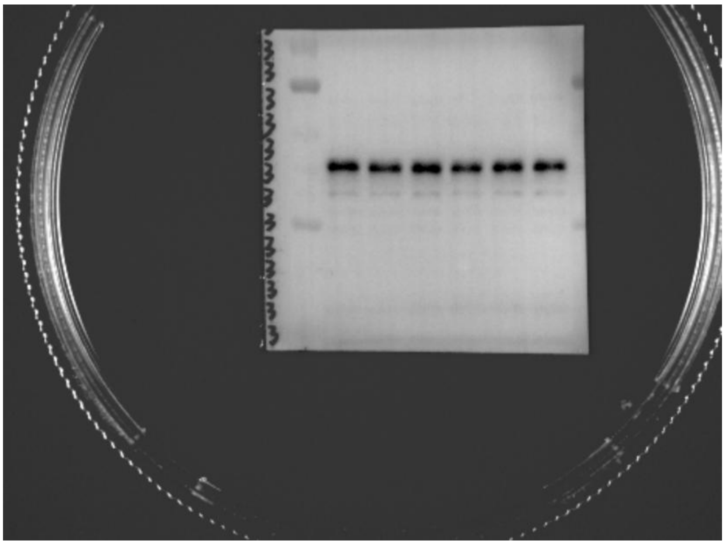

α-SMA

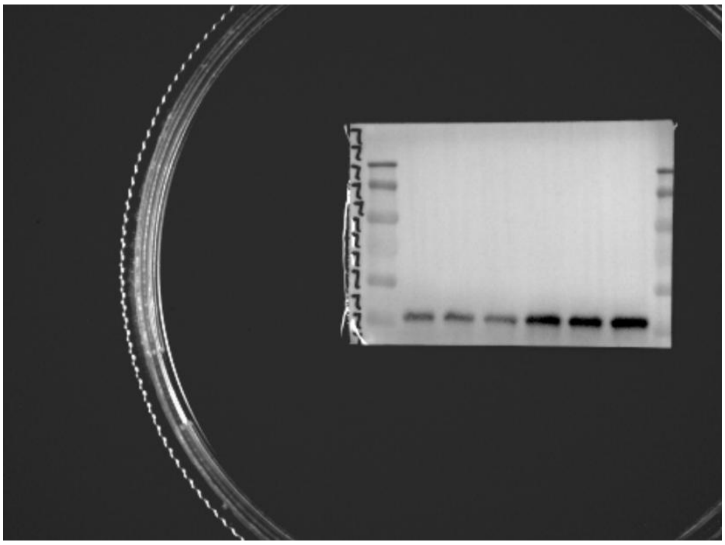

GAPDH

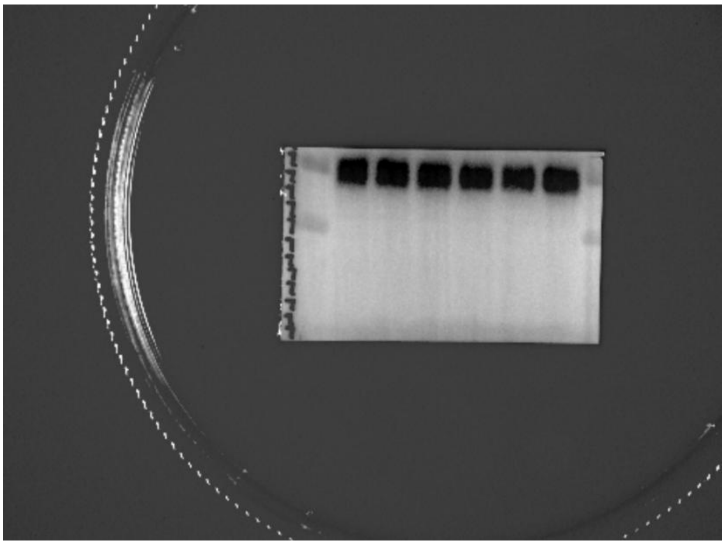

Vimentin

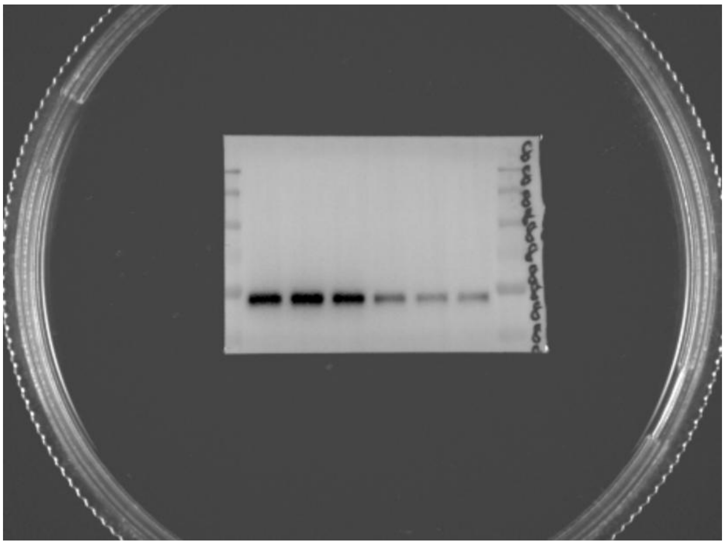

GAPDH

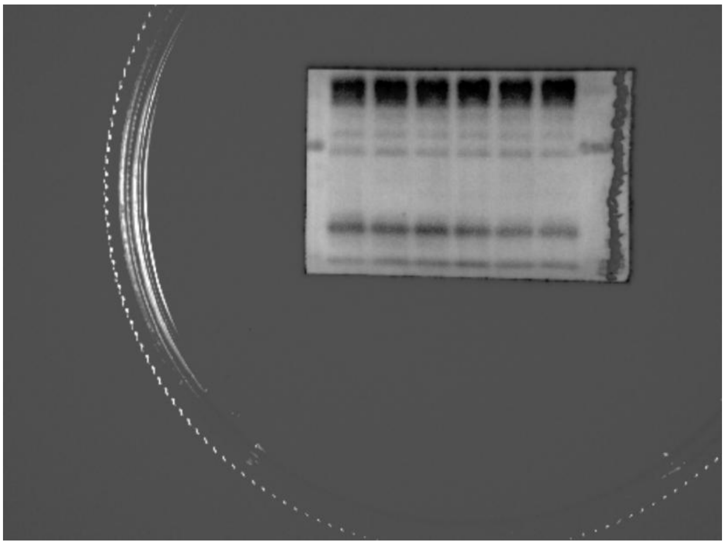

Supplement: Supplementary file 1 — Supplementary Information 1. [file 41598_2024_55408_MOESM1_ESM.pdf]
